# Reply: neutral transcriptome rewiring promotes quantitative disease resistance evolvability at the species level

**DOI:** 10.1093/plcell/koag068

**Published:** 2026-03-16

**Authors:** Florent Delplace, Mehdi Khafif, Adelin Barbacci, Remco Stam, Sylvain Raffaele

**Affiliations:** Laboratoire des Interactions Plantes-Microbes Environnement (LIPME), INRAE, CNRS, Université de Toulouse, Castanet-Tolosan 31326, France; Laboratoire des Interactions Plantes-Microbes Environnement (LIPME), INRAE, CNRS, Université de Toulouse, Castanet-Tolosan 31326, France; Laboratoire des Interactions Plantes-Microbes Environnement (LIPME), INRAE, CNRS, Université de Toulouse, Castanet-Tolosan 31326, France; Department of Phytopathology and Crop Protection, Institute of Phytopathology, Faculty of Agricultural and Nutritional Sciences, Christian-Albrechts-University, Kiel 24118, Germany; Laboratoire des Interactions Plantes-Microbes Environnement (LIPME), INRAE, CNRS, Université de Toulouse, Castanet-Tolosan 31326, France

Dear Editor,

We thank Drs. Balint-Kurti and Wagner for their thoughtful critique of our study (Delplace et al. 2025) and welcome the opportunity to clarify our methodological choices, interpretations, and conclusions. Their letter raises questions on 3 points: (1) the relationship between transcriptional responses and quantitative disease resistance (QDR), (2) the limitations of single-timepoint analyses, and (3) the evolutionary interpretation of transcriptome-phenotype maps. Many of these concerns touch on considerations relevant to transcriptomic and plant pathology research more broadly. We therefore view this correspondence as an excellent opportunity to discuss shared challenges and advance perspectives for our field as a whole. We address each major point in turn below.

## Relationship between transcriptional responses and disease resistance

Balint-Kurti and Wagner express concern about evidence for a causal link between the transcriptional response and disease resistance phenotype being weak in our study, with 3 underlying arguments: (1) Genes contributing to resistance or susceptibility likely represent a small, undetermined proportion of differentially expressed genes; (2) correlation between gene expression and disease susceptibility is generally weak in our study; and (3) this limits the use of our dataset to predict QDR. These aspects were discussed in our original manuscript (paragraphs “Correlation between gene expression and disease susceptibility is marginal at the species level”, “How predictable is QDR?”, and “What is the contribution of the variable transcriptome to A. thaliana QDR?”), and we welcome the opportunity to elaborate further here.

### What proportion of differentially expressed genes contributes to disease resistance?

Comparative gene expression studies can be leveraged notably to mine for genes contributing to a phenotype of interest or to infer system-level properties leading to the emergence of complex traits. The latter was our main focus in Delplace et al. (2025). Nevertheless, the potential for assigning QDR functions to genes is of practical interest and worth further discussion. To identify candidate loci contributing to complex traits, testing associations between gene expression and resistance variation across accessions is a standard approach in quantitative genetics. It is widely used in GWAS and QTL mapping studies (Atwell et al. 2010; Wray et al. 2013). The omnigenic model of complex traits (Boyle et al. 2017; Liu et al. 2019) provides a useful framework for interpreting such associations. It posits that most genes expressed in trait-relevant tissues can contribute to heritability, either through direct effects or indirect regulatory influences. Importantly, several core DEGs from our study, such as the camalexin biosynthesis gene *PAD3* (Stotz et al. 2011), the abscisic acid transporter gene *ABCG40* (Sucher et al. 2020), and the leucine-rich repeat receptor gene *RLP30* (Zhang et al. 2013) have well-established roles in resistance to *S. sclerotiorum*. Besides, natural variation in gene expression has been associated with QDR in several pathosystems (Badet et al. 2019; Zhu et al. 2024), supporting a functional link between transcriptional reprogramming and disease resistance outcomes.

We reported that core DEGs were enriched in gene ontology (GO) terms related to response to infection (Delplace et al. 2025). To determine the proportion of DEGs with a known function in response to infection, we established a list of 74 infection-related GO terms (Table S1) and identified 2,380 genes harboring these GOs (Table S2). Among infection-related genes, 1,655 (69.5%) were DEG in at least 1 accession. Genes upregulated in all 23 accessions (core up-regulated DEGs) included 19% of infection-related genes, representing a 2.21-fold enrichment relative to the genome content (Pearson chi-squared 149.25, *P*-value 2.5E−34). Many of these genes are known defense gene-homologs, yet some might negatively contribute to plant immunity and act as susceptibility factors. Because of QDR polygenic nature, many QDR genes may not be annotated as infection-related yet, leading to underestimating the real extent of infection-related genes among DEGs. In other pathosystems, functional validation of numerous mutants has revealed that transcriptional reprogramming plays a major role in conferring quantitative disease resistance (AbuQamar et al. 2006; Vergne et al. 2010; Schweiger et al. 2016). Our preliminary validation of 2 mutants corresponding to novel QDR-associated genes (Delplace et al. 2025; Fig. S13) supports this link between differential expression and QDR phenotype. Systematic functional testing will be required to establish causality on the global scale. Currently, the convergence of transcriptional, genetic, and phenotypic data provides a strong foundation for interpreting transcriptome variation as a biologically meaningful substrate for QDR evolution and constitutes a solid basis for future investigation.

### To what extent does gene expression correlate with disease susceptibility?

**Figure 1:**
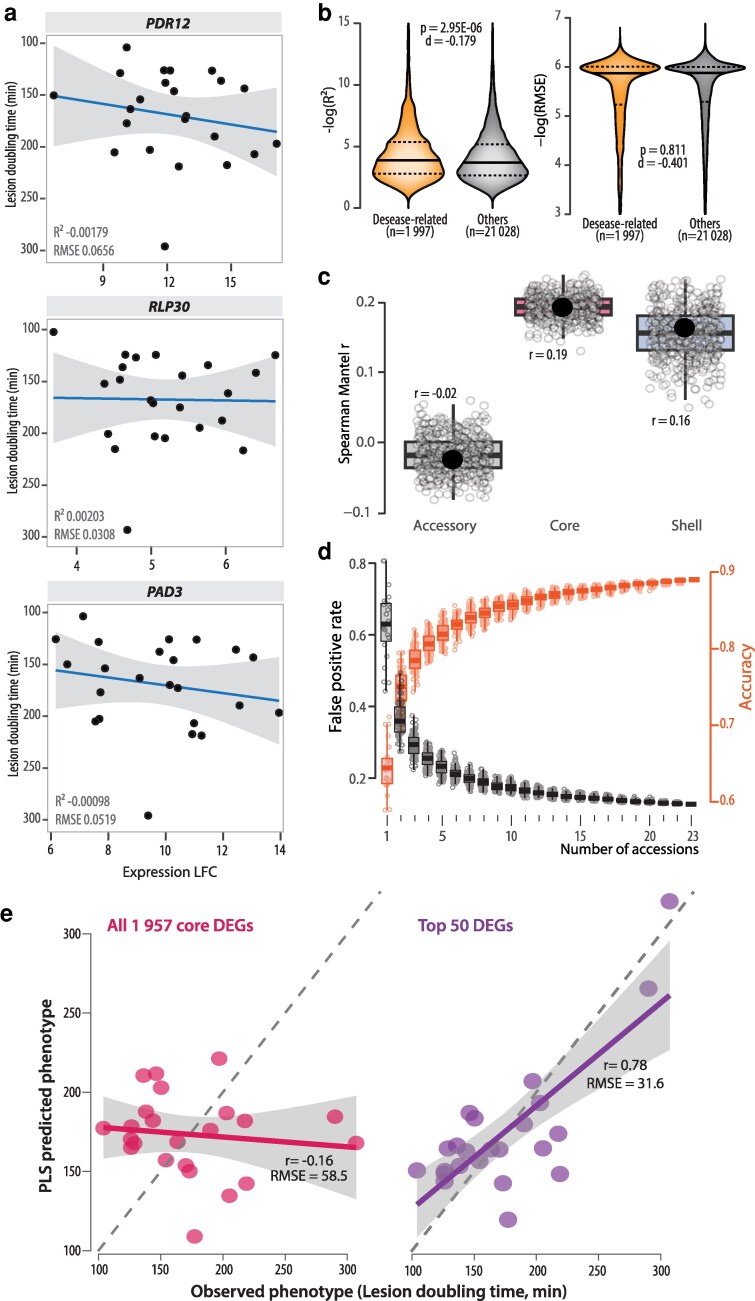
Relationship between gene expression and role in disease resistance. a) Correlation between log2 fold change (LFC, *x* axis) and disease resistance (lesion doubling time in minutes, *y* axis) in 23 accessions for 3 genes with well-established role in disease resistance against *S. sclerotiorum*. The blue lines show linear regression of the data with *R*^2^ and *P*-value labeled, and the 95% confidence interval shown as a gray area. b) Distribution of R^2^ and root mean squared error (RMSE) for 1,997 disease-related genes (with disease related gene ontology) and other genes. Dotted lines show first and third quartiles, the plain lines show mean of each distribution. *P*-values from Student *t* test and effect size estimated as Cohen *d* are labeled. c) Mantel correlation between gene expression and lesion doubling time phenotypes of 23 accessions. Plain dots show correlation for full gene sets, empty dots show random samples of 500 genes taken from each gene set. d) Measures of error in assigning a role in disease resistance to upregulated genes as a function of the number of accessions analyzed. Values for 2 to 22 accessions were determined based on random sampling (100 replicates) of accessions. Boxplots show first and third quartiles (box), median (horizontal line), and the most dispersed values within 1.5 times the interquartile range (whiskers). e) Comparison between observed disease resistance phenotypes (*x* axis) and phenotypes predicted by partial least square (PLS) regression on gene expression (*y* axis) for all core genes (left) and top50 core genes (right). The gray areas indicate 95% confidence intervals; the dotted lines indicate *X* = *Y*. Pearson correlation and RMSE between observed and predicted values are labeled.

The omnigenic view of complex traits suggests that most genes responding to inoculation contribute to QDR to some extent. Nevertheless, the transcriptional response of each single gene also depends on the overall genetic background of each accession (Einspanier et al. 2025b). In addition, many post-transcriptional regulation layers may complicate the relationship between individual gene expression and the phenotype they are involved in. As a result, it is relatively unlikely that the expression of a single QDR gene would scale linearly with disease resistance. Instead, complex multi-factorial relationships between gene expression and QDR phenotypes are expected in this context. For instance, we show here that the expressions of *PDR12*, *RLP30*, and *PAD3*, which have established roles in QDR against *S. sclerotiorum* (Stotz et al. 2011; Zhang et al. 2013; Sucher et al. 2020), did not significantly correlate with disease lesion development (Fig. 1a). Despite limits due to the polygenic nature of QDR and genotype dependency of individual gene expression (Delplace et al. 2025), tested whether a subset of genes would show an expression linearly correlated with QDR phenotypes. This identified 32 genes that may be conserved regulators of QDR, as they may be involved in QDR across multiple genetic backgrounds. To broaden this observation, we extracted correlation statistics (R^2^ and RMSE) for 1,997 genes with an infection-related GO. These genes had a significantly higher R^2^ than other genes (average 0.051 and 0.044, Student *t* test *P*-val 2.95E−06), suggesting a trend for better correlation with QDR phenotype (Fig. 1b); however, this was negligible in effect size assessment (Cohen d −0.179). There was no significant difference in RMSE for genes with infection-related GOs and other genes (*P*-value = 0.81; Fig. 1b). Next, we analyzed the number of genes with infection-related GOs among the 150 genes with the highest variance contribution in the principal component analysis (PCA) reported by Delplace et al. (2025). We found 23 genes with infection-related GOs, representing 15.3% of the 150 genes with the highest variance contribution, and a ∼1.72-fold enrichment relative to all DEGs (chi-squared test *P*-value = 5.75E−03). This indicates that the PCA did not represent statistical noise in the data but accurately reported on biologically relevant genes with a clear association with infection-related processes.

The modest correlation strengths for individual genes overall could reflect either weak biological relationships or the existence of multiple regulatory strategies that achieve similar resistance outcomes through different molecular routes, a many-to-one mapping between genotype and phenotype (Paaby and Rockman 2013). In polygenic traits with regulatory flexibility, increasing the number of genotypes sampled may paradoxically decrease average correlation strength if lineages have independently evolved alternative defense strategies, even as individual gene-phenotype associations remain biologically meaningful within specific regulatory contexts (Gibson 2012). Consistent with this framework, Delplace et al. (2025) identified 8 gene coexpression modules (166 to 1,281 genes each) significantly correlated with the QDR phenotype, including 1 enriched for immune-related genes. However, as mentioned in the Results section, the correlations were modest (|R^2^| = 0.12 to 0.21, *P* < 0.05; using Spearman permutation test [n = 9,999]) and were mainly driven by changes in a single accession. These modules represent coordinated gene sets rather than isolated loci, as expected for highly polygenic traits where numerous small-effect loci contribute additively (Hill 2010; Visscher et al. 2017; Hoheneder et al. 2023; Einspanier et al. 2025b). As a complementary approach, we tested here the Mantel correlation between expression of core, shell, and accessory genes and the phenotypes of accessions. The Mantel test measures the correlation between 2 distance matrices on the same individuals or populations and allows determining whether transcriptomic distances reflect phenotypic distances. The strongest correlation was observed for core genes (r = 0.19), slightly higher than for shell genes (r = 0.16) and substantially higher than for accessory genes (r = −0.02) (Fig. 1c). To ensure that these results were not merely due to differences in the number of genes per category, we performed a bootstrap analysis by randomly sampling 500 genes per category and recalculating the Mantel correlation coefficient. The trend observed with all genes of each category was preserved (Fig. 1c). These analyses indicate that, in spite of limited expression-phenotype correlations, expression and phenotype are not independent and that the relationship between core genes and phenotypes is stronger.

### Can we predict QDR?

The questions raised here involve 2 distinct aspects: (1) whether differential gene expression is predictive of a function in QDR, and (2) whether the correlation of expression and phenotype is predictive of a function in QDR. We first tested here the hypothesis that knowledge on upregulated genes helps predict genes with a function in defense. For this, we calculated the false positive rate and accuracy of identifying the 2,380 *Arabidopsis* genes with infection-related GO based on upregulation in 1 to 23 accessions (Fig. 1d). False positive rate (wrongly predicting an infection-related gene if upregulated) decreased from 80.6% for genes upregulated in Ang-0 or Nok-3 to 12.7% for genes upregulated in 23 accessions. In average, the accuracy of identifying an infection-related gene based on upregulation increased from 64.5% for genes upregulated in 1 accession to 89% for genes upregulated in 23 accessions. This highlights the benefit of transcriptome studies in multiple accessions and suggests that core DEGs are promising candidates for future functional studies on QDR.

Second, we expanded on our tests for a link between gene expression and QDR phenotype. As mentioned previously, the polygenic nature of QDR makes it unlikely that the expression of a single gene would scale linearly with disease resistance, a prediction that has rarely been challenged, and that our data support. By contrast, machine learning (ML) proved more powerful in capturing complex nonlinear patterns of gene expression associated with *A. thaliana* QDR against *B. cinerea* (Sia et al. 2025). In their study, Sia et al. reported that ML models trained on *A. thaliana* transcriptome were able to accurately predict disease outcome, including after inoculation by *S. sclerotiorum*. This suggests that gene expression and QDR phenotypes are linked through complex relationships not detectable through classical correlative approaches. To verify that this conclusion applies to 23 accessions dataset, we used partial least square (PLS) regression to predict the average lesion doubling time of each accession. This approach complements the dimensionality reduction by PCA used in (Delplace et al. 2025) through the construction of latent variables (as linear combinations of gene expression) for dimensional reduction. We tested the predictive ability of the core transcriptome using PLS models with leave-1-out cross-validation. When all core genes are included in the PLS model, the correlation between observed and predicted phenotypes remains low (Spearman r = −0.16; Pearson r = −0.12), indicating that most transcriptomic variation is neutral with respect to the phenotype, consistent with the concept of neutral rewiring. However, a PLS model based on the 50 core genes most correlated with the phenotype markedly improved predictions, with Spearman r = 0.48, Pearson r = 0.78, and RMSE reduced from 58.5 to 31.6 (Fig. 1e). To assess the relative importance of each gene in the model, we extracted the loadings from the first PLS component, which represents the weight of each gene in constructing the latent variable (Table S3). Genes with higher absolute loadings contribute more strongly to the phenotype prediction. Forty-six of these 50 top contributing core genes showed loadings ranging from 0.103 to 0.179, indicating relatively balanced contributions across the selected gene set, with no single gene dominating the predictive model. This result demonstrates that plant gene expression and disease resistance to *S. sclerotiorum* are not independent and that a fraction of the core transcriptome is sufficient to achieve good prediction accuracy.

## Temporal dynamics and the broad-spectrum nature of QDR

Second, Balint-Kurti and Wagner raise awareness on the scope of our analyses, that derive from a single plant pathogen interaction (*A. thaliana* inoculated with *S. sclerotiorum*) at a single time point (24 h post inoculation). This limitation was explicitly acknowledged in our manuscript, and we agree that defense responses can be highly dynamic over time. We discussed this property in our article (section “What is the contribution of core DEGs to *A. thaliana* QDR phenotype?”). We take advantage of this letter to further discuss the diversity of transcriptional responses to pathogens across time and interactions.

### How representative is the 24-hpi timepoint?

**Figure 2:**
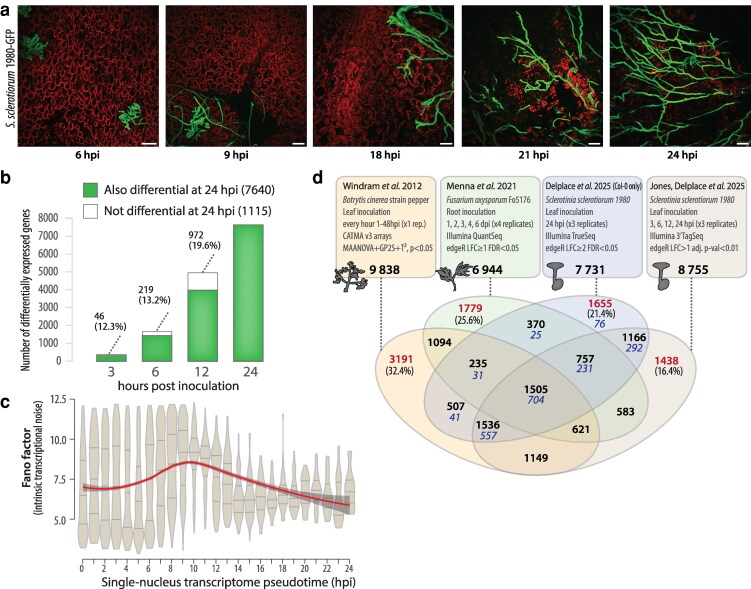
Diversity of *A. thaliana* responses to fungal pathogens across time and pathosystems. a) Representative confocal micrographs of *A. thaliana* leaves inoculated by *S. sclerotiorum* expressing GFP. Pictures were taken at 6, 9, 18, 21, and 24 hpi at the edge of visible disease lesions. Scale bar = 100 µm. b) Number of differentially expressed genes identified in a time course of *A. thaliana* inoculation by *S. sclerotiorum* (Jones et al. 2026), highlighting DEGs not detected at 24 hpi (white bar). Number of genes and proportion of DEGs detected at each time point are labeled. c) Measure of intrinsic transcriptional noise along pseudotime in a single-nucleus RNA-seq analysis of *A. thaliana–S. sclerotiorum* interaction (Jones et al. 2026). Kernel probability of the data is shown in gray with median and quartile values shown as horizontal gray lines. Red lines show loess local regression, dark gray area shows and 95% confidence intervals. Reproduced from Jones et al. (2026, Figure 5A). d) Distribution of differentially expressed genes identified in 4 studies of *A. thaliana* transcriptional responses to fungal pathogens. The design of each study and the total of DEGs identified is indicated in rounded boxes. DEGs unique to 1 study are labeled in red with % indicating the proportion they represent of all DEGs identified in the corresponding study. The number of cores DEGs from Delplace et al. (2025) is labeled in blue italics.

Based on a detailed time course of *Botrytis cinerea* inoculation of *A. thaliana* leaves, Windram et al. (2012), cited by Balint-Kurti and Wagner in their letter, report that “the majority of changes in gene expression have occurred by 24 HAI when the pathogen has penetrated the leaf epidermis but only very small, localized lesions are present”. This rationale motivated our focus on the 24-hpi timepoint. Our previous observations indicated that at 24 hpi, *S. sclerotiorum* has established intercellular infection, begun tissue colonization and disease lesions are actively growing (Barbacci et al. 2020). This makes 24 hpi a biologically relevant stage for assessing both transcriptional responses and QDR phenotypes across genotypes. For a precise account of the dynamics of the infection process, we inoculated *A. thaliana* leaves with a strain of *S. sclerotiorum* constitutively expressing GFP (Badet et al. 2017) and examined tissue colonization with confocal microscopy (Fig. 2a). At 6 and 9 hpi, *S. sclerotiorum* growth was mostly at the leaf surface with a few infection cushions in contact with plant cells. Invasive growth involving intercellular fungal hyphae became apparent around 18 hpi, with limited cell death symptoms. Invasive growth was well established with extensive dead cell patches at 21 and 24 hpi.

Recent studies emphasized that while early responses (0 to 12 hpi) are critical for determining infection outcomes (Liu et al. 2024), later responses (12 to 24 hpi) reflect the integrated output of defense programs and their effectiveness against pathogen colonization (Lewis et al. 2015; Einspanier et al. 2025a). We took advantage of a recent study (Jones et al. 2026) in which we determined *A. thaliana* transcriptional responses at 3, 6, 12, and 24 hpi with *S. sclerotiorum* to test how many transcriptional changes occurring during the interaction are captured at 24 hpi (Fig. 2b). This study identified 375, 1,660, 4,958, and 7,640 genes differentially expressed respectively at 3, 6, 12 and 24 hpi. Among those, only 46 (12.3%), 219 (13.2%), and 972 (19.6%) were differential at 3, 6, and 12 hpi, respectively, but not at 24 hpi. Overall, 87.3% of DEGs at earlier time points were also differential at 24 hpi. Using single-nucleus RNA-sequencing, we showed that intrinsic transcriptional noise was high before 16 to 18 hpi, at which time cells involved in defense response clearly committed to transcriptional reprogramming (Fig. 2c). Therefore, by sampling at 24 hpi, we captured the stabilized transcriptional state resulting from early decision-making processes, providing a phenotypically informative snapshot of *A. thaliana*-*S. sclerotiorum* interaction.

### Are transcriptional responses to *S. sclerotiorum* specific to 1 pathogen?

Our study indicates that QDR in the *Arabidopsis*-*Sclerotinia* pathosystem integrates multiple conserved defense pathways. Among activated pathways, camalexin biosynthesis supports a broad-spectrum model of QDR. Indeed, this pathway, part of the core DEGs (differentially expressed in all accessions), is induced not only by *S. sclerotiorum* but also by phylogenetically diverse pathogens such as *Botrytis cinerea* (Stotz et al. 2011), *Colletotrichum higginsianum* (Hiruma et al. 2010) and *Alternaria brassicicola* (Thomma et al. 1999). Such evidence suggests that shared genetic toolkits underpin host responses to distinct pathogens, in good agreement with studies reporting the identification of loci conferring quantitative disease resistance to multiple diseases (Martins et al. 2019; Zhu et al. 2024). Besides, we noted that several time course studies reported 1 “major shift in gene expression” (Windram et al. 2012), for instance, around 24 hpi with *B. cinerea* (Windram et al. 2012) or at 3 days post-inoculation with *Fusarium oxysporum* (Menna et al. 2021). To determine the extent to which the nature of transcriptional changes differ in these interactions and test whether our analysis identified potential multiple disease resistance (MDR) genes, we compared the identity of DEGs reported in 4 studies (Fig. 2d). These studies all deal with fungal pathogens that exhibit a prevalent necrotrophic phase and are therefore not meant to represent the full range of pathogenic interactions that Arabidopsis can encounter in Nature. Nevertheless, they cover a diversity of colonization strategies with root (*F. oxysporum*) and foliar inoculations (*B. cinerea* and *S. sclerotiorum*), sampling strategies (single time point or time course), gene expression quantification methods (Microarrays, RNA-sequencing, 3′Tag-Sequencing) and differential analysis strategies. Despite these very different study designs, no more than one third of DEGs were unique to 1 study (32.4% in Windram et al. 2012). Out of 1,957 core DEGs identified in Delplace et al. (2025), only 76 (3.9%) were not differential in at least 1 other study. Core DEGs from Delplace et al. (2025) represented 46.8% (704 genes) of DEGs common to all 4 studies and may therefore provide a list of broad-spectrum QDR gene candidates. Together, these comparative analyses suggest that our conclusions may not be highly specific to a single disease and timepoint. It should be noted, however, that which genes actually contribute to disease resistance in each of these interactions remains to be determined.

Because QDR leverages conserved cellular functions, it tends to exhibit broad-spectrum efficiency, mitigating disease severity by activating common genetic and metabolic pathways (Delplace et al. 2022). Critically, although core defense modules are conserved across genotypes, natural variability in *A. thaliana* and tomato QDR against *S. sclerotiorum* reveals that specific defense functions have diverged (Delplace et al. 2025; Einspanier et al. 2025b). While our findings are derived from a single pathosystem, the recurrent activation of shared defense modules across diverse pathogens suggests that QDR may operate through evolutionarily conserved, modular networks. The variability observed within *A. thaliana* serves as proof of concept, demonstrating the plasticity of these mechanisms at the species level. To distinguish core, conserved features of QDR from lineage-specific adaptations, future comparative studies across multiple host–pathogen systems will be essential.

## Transcriptome-phenotype maps as a framework for understanding QDR evolvability

Finally, Balint-Kurti and Wagner argue that our finding of a navigable transcriptome-QDR phenotype map is “inevitable” and that other traits should be considered to conclude on the evolvability of QDR. We made clear in our article that our transcriptome-phenotype maps are not traditional fitness landscapes (Poelwijk et al. 2007) but rather QDR phenotype landscapes in gene expression space, a distinction that Balint-Kurti and Wagner cautiously report at the beginning of their comment. This comment provides an opportunity to address what limits the evolvability of QDR, that we wish to discuss hereafter.

### What are the determinants of QDR evolvability?

We adapted methods from evolutionary genetics (de Visser and Krug 2014; Greenbury et al. 2016) to estimate the navigability of transcriptional states, that is the proportion of expression space accessible via mutational steps without substantial resistance loss. The neutral network concept predicts that genotypes connected by such neutral paths can explore novel transcriptomes without QDR penalties, thereby promoting evolutionary potential under pathogen pressure (Greenbury et al. 2016, 2022). Our analysis revealed that the transcriptomic landscape of QDR is highly navigable. Indeed, 15 accessions could each traverse more than 3% of the transcriptome space with no loss of resistance, and most accessions showed intermediate resistance levels that allowed extensive neutral exploration. This indicates that multiple transcriptional configurations can produce similar resistance outcomes, exemplifying a many-to-one genotype–phenotype mapping (Paaby and Rockman 2014). This pattern is consistent with QDR being predominant in natural populations (Corwin and Kliebenstein 2017). It also suggests that transcriptional plasticity provides a broad substrate for evolving resistance, since high navigability would enable populations to respond to diverse or evolving pathogen pressures without falling into low QDR valleys.

**Figure 3:**
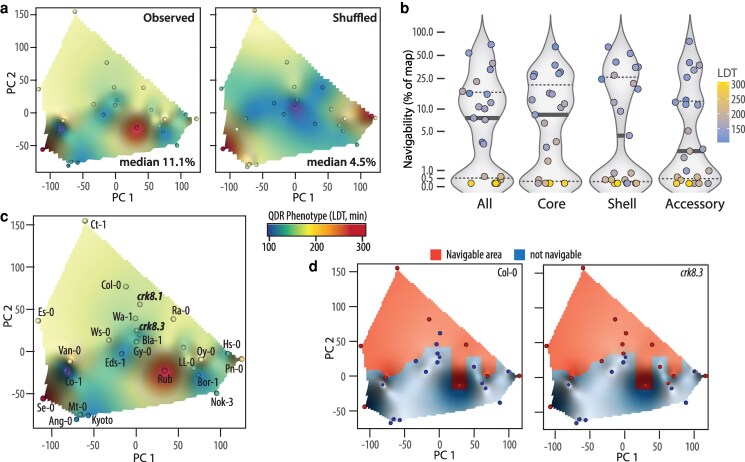
Navigability of transcriptome-phenotype maps and the evolvability of QDR. a) Transcriptome-resistance maps showing *A. thaliana* accession proximity in the transcriptome space in response to *S. sclerotiorum* inoculation. The *x* and *y* axes represent principal components (PC) 1 and 2, the color gradient represents disease resistance as indicated by the lesion doubling time (LDT) in minutes, from more susceptible (blue) to more resistant (red). The comparison between experimentally determined map (left, Delplace et al. 2025) and an artificial map in which transcriptome-phenotype associations have been shuffled (right) illustrate topologies leading to reduced overall navigability (median labeled, as % of map surface). b) Distribution of navigability for 23 *A. thaliana* accessions in transcriptome-phenotype maps generated with all, core, shell, and accessory genes (Delplace et al. 2025). Dotted lines show first and third quartiles; plain line shows mean value. c) Transcriptome-phenotype map on which the transcriptome and phenotype of *crk8.1* and *crk8.3* (Macquet et al. 2025) in response to *S. sclerotiorum* have been projected. d) Comparison of Col-0 wild type (left) and *crk8.3* mutant (right) navigable area on *A. thaliana* transcriptome-resistance map. Red dots correspond to natural accessions with higher LDT (more resistant).

QDR being governed by multiple genes of weak phenotypic effect, a broad range of mutations could theoretically improve this trait (increasing transcriptome-phenotype map navigability), but many mutations could also impair this trait. The resulting effect on QDR evolvability is likely not only the result of its polygenic nature, but also of the architecture of QDR gene networks. By shuffling QDR phenotypes in the transcriptome space covered in our analysis, we identified artificial maps with strongly reduced navigability (Fig. 3a), supporting the transcriptome-phenotype link as a key determinant of navigability. Besides, we reported that navigability was generally higher on the core DEG transcriptome map (Fig. 3b) and that disease resistance correlated with navigability on the core DEG map, but not on the whole-transcriptome and shell DEG maps (Delplace et al. 2025, Fig. 6). This partitioning shows that navigability is not a uniformly neutral property of the transcriptome. Rather, a conserved core network acts as a regulatory backbone under stronger selective constraint (Delplace et al. 2025, Fig. 3F, G), while peripheral transcriptional variation is more freely explored or exploited for alternative defense strategies (Rockman 2012). Consequently, major alterations to the core DEG network likely increase significantly the ruggedness of the transcriptome-QDR map and therefore reduce navigability. The specific correlation between core DEG navigability and resistance implies that constraints and neutralities vary across functional gene sets, which is consistent with a polygenic architecture where different gene subsets experience different selection pressures (Hill 2010).

### On the impact of tradeoffs on the evolution of *A. thaliana* response to *S. sclerotiorum*

As noted by Balint-Kurti and Wagner, the transcriptome-phenotype map we propose does not provide any direct information on the impact of QDR-related genes on other traits. However, the evolvability of QDR is not treated in isolation from other traits in this map, since the expression patterns we measured have been shaped by constraints on all plant traits during diversification of the species. Thus, we can infer that tradeoffs only weakly constrained the global evolution of *A. thaliana* transcriptional responses to *S. sclerotiorum*, possibly because navigability of the transcriptome-QDR map is high. Indeed, along neutral paths, alterations to gene expression that may be driven by tradeoffs in favor of traits other than QDR are predicted to alter the QDR phenotype only weakly. Reciprocally, neutral paths might support the frequent exaptation into QDR of genes with other functions (Macquet et al. 2022; Einspanier et al. 2025b).

Recent work shows that optimal fitness often requires integrated coordination of growth and defense rather than a strict antagonism between them (Kliebenstein 2016; Züst and Agrawal 2017). We previously reported that expression variation at the *CRK8* locus underlies a tradeoff between disease resistance to *S. sclerotiorum* and seed germination on NaCl (Macquet et al. 2025). Using RNA-seq of *crk8.1* and *crk8.3* mutants 24hpi following *S. sclerotiorum* inoculation, we positioned these genotypes in the principal component plane obtained for *A. thaliana* 23 accessions (Fig. 3b). The *crk8.1* allele, which did not significantly alter disease resistance, was located between Col-0 and Ra-0 on the PCA plane. The *crk8.3* allele, which caused a 14% reduction in QDR, is located close to Bla-1 and Gy-0 accessions that show 23% and 15% reduction in QDR, respectively, compared with Col-0. Considering their position on the PCA plane and their respective QDR phenotype, the *crk8.1* and *crk8.3* only caused minimal alterations to the transcriptome-phenotype map. Next, we compared navigability for Col-0 and *crk8.3*, corresponding to the area of the transcriptome-phenotype map that can be reached through increasing QDR phenotype (Fig. 3c). The *crk8.3* mutation led to a 1.71% increase in navigability. This particular example illustrates how tradeoffs may translate into the transcriptome-QDR analysis framework. Tradeoffs may also lead to more complex transcriptome alterations that cannot be captured by the current transcriptome-QDR map, emphasizing the need to expand our current exploration of this transcriptome space.

## Conclusion

The dataset reported in Delplace et al. (2025) provides an extensive transcriptomic resource capturing natural variation in *A. thaliana* QDR against *S. sclerotiorum*. Yet, it represents a small part of the complex landscape of plant disease resistance. Using a transcriptome-phenotype map model with assumptions, we inferred hypotheses on the evolution of QDR, which should be treated with caution given the limitations of all model-based approaches. Similarly, knocking out a gene, observing a phenotype and inferring biological function relies on a model of what the gene product might do at the molecular, cellular, and organ level. This model is generally implicit, and yet often extrapolated as proof to validate other models. Building on reasonable assumptions, models enable testing more elaborate hypotheses and achieving a deeper understanding of cells functioning.

Sia et al. achieved similar QDR phenotype prediction accuracy using expression of diverse gene sets, suggesting that the predictors likely reflect important pathways or network modules rather than specific gene effects (Sia et al. 2025). This complexity is inherent to polygenic traits and highlights the value of multi-omics approaches, which can reveal convergent functional modules and mechanistic logic underlying complex plant traits beyond what single-layer analyses capture. Combining omics approaches across spatial and temporal dimensions could help identify these functional modules, their regulatory logic, and their context-dependent activation patterns. Experimentally validating polygenic traits requires moving beyond single-gene knockouts toward system-level perturbations and predictive modeling. Multiplex genome editing could be leveraged to perturb multiple network components simultaneously to assess their collective contribution to resistance. Critically, such experiments should be guided by network models that predict which gene combinations are functionally redundant, synergistic, or antagonistic. Our evolutionary genetics framework, showing that neutral transcriptional rewiring maintains evolvability, suggests that many individual mutations may have minimal effects precisely because networks are robust to single perturbations. Additionally, machine learning approaches trained on multi-omics datasets can predict resistance phenotypes from molecular profiles and guide the selection of key genetic mechanisms (Sia et al. 2025). High-resolution phenotyping, deep sampling, and the use of an evolutionary framework such as suggested in Delplace et al. (2025) could improve both accuracy and transferability of prediction models. A broader exploration of plant-pathogen interactions, including multi-omics approaches, will thus be needed to provide us with sets of genes playing a key role in plant defense responses.

Gene-mining studies have practical and conceptual interests, including the design of genetic strategies for the management of diseases, and extrapolation on the function of related molecular components. Beyond such targeted aims, large datasets also offer the opportunity to gain understanding of the functioning of cells and genomes as wholes, as complex systems, with properties that do not exist at the gene and lower organization levels (emergent properties). Despite the acceleration of data generation, this perspective remains underexplored. We showed that the quantitative nature of the defense phenotype we studied is not much the result of quantitative variation in the expression of single genes (except for 32 gene candidates) but rather emerges at the transcriptome level, through coordinated differential expression of multiple sets of genes. Reciprocally, large-scale transcriptional reprogramming can occur without proportional changes in resistance, highlighting the importance of regulatory context over individual gene effects. By combining transcriptomics and evolutionary genetics concepts, we explored evolvability of *A. thaliana* responses to *S. sclerotiorum*, that is the ability of defense responses to incorporate adaptive genetic diversity and evolve through natural selection.

Understanding emergent cellular properties requires conceptual frameworks that extend beyond gene-centric views, a transition that may require time for the field to embrace. Together, our findings lay a foundation for understanding how transcriptional variation underpins quantitative immunity and its evolvability. There are many significant (and highly interesting) outstanding questions pertaining to how QDR is regulated and evolved. We look forward to seeing these exciting questions being addressed by the field, and emerging properties of the plant immune system being explored in the coming years.

## Supplementary Material

koag068_Supplementary_Data

## Data Availability

The are no new original data associated with this article.
