# Supplementary material for: Reply: neutral transcriptome rewiring promotes quantitative disease resistance evolvability at the species level
Source: Plant Cell. 2026 Mar 16;38(5):koag068. doi: 10.1093/plcell/koag068 (PMC13221639; doi:10.1093/plcell/koag068)
Supplement: koag068_Supplementary_Data [file koag068_supplementary_data.zip › FigS13.pdf]

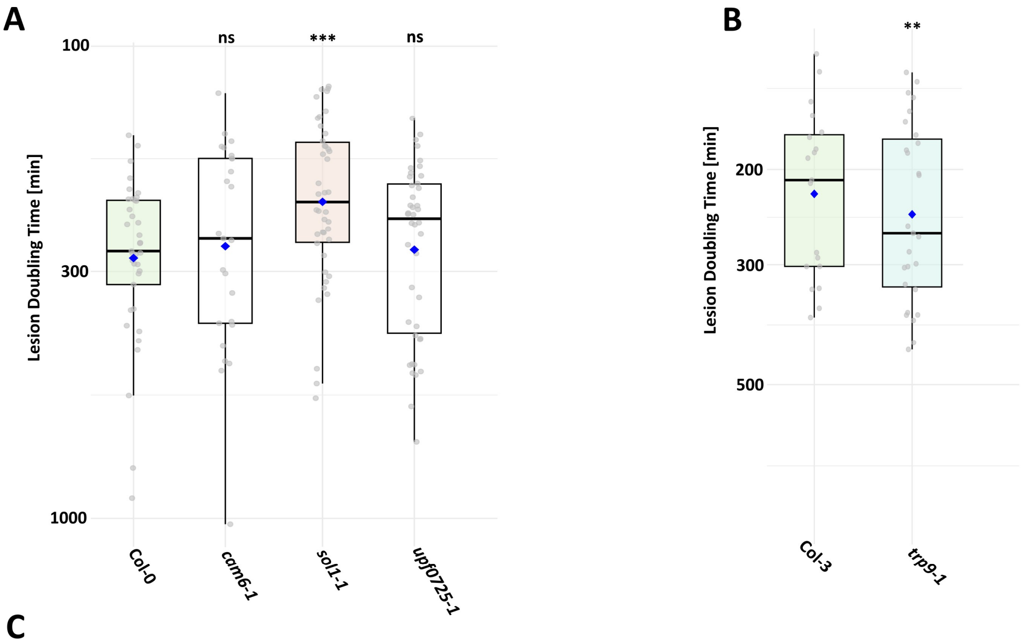

C

Gene	Mutant lines	NASC number	Genetic background	T-DNA insertion location	Mean Lesion Doubling Time	Number of experiments	Number of leaves	P-value	Forward primer	Reverse primer
AT5G21274	*cam6-1*	N656633	Col-0	Exon	238.2	2	25	0.08367	AGCCAGAATCAAAACCCTAGC	TTTTTGCTTATACATATCCGTTTCTG
AT3G22760	*sol1-1*	N861176	Col-0	Intron	199.2	3	42	8.62e-05	AATCCAACTATTTGCAACCATG	CTTTATGAGAAACCGCGTGAG
AT4G29550	*upf0725-1*	N677698	Col-0	Exon	249.3	3	42	0.49013	GTCTTGTCTCCGAACTTGGTG	GTGACATTGTTGGCATTTGTG
	Wild-Type	-	Col-0	-	263.6	3	37	-		
AT1G33400	*trp9-1*	N866530	Col-3	Exon	225.8	2	27	0.006884	CATGTATCACAGAAGCTTCTTCG	GAATTCGAAACGAAAGTGTGC
	Wild-Type	N908	Col-3	-	186.3	3	20	-		
									LB1_SAIL	GCCTTTTCAGAAATGGATAAATAGCCTTGCTTCC
									LBb1.3_SALK	ATTTTGCCGATTTCGGAAC

Supplemental Figure S13: Two mutants for two genes exhibited a significant alteration in their response to *Sclerotinia sclerotiorum*.

A. The lesion doubling time (LDT, y-axis; log scale) in minutes represents the susceptibility of genotype 24 hours after inoculation with S. sclerotiorum. Measurements were obtained from n = 25 to 42 leaves across two to three experiments (see Table in C). LDT was measured in Columbia-0 (Col-0), the wild-type genotype, and in three mutants corresponding to three genes. Boxplots display the first and third quartiles (box), the median (thick line), and the mean LDT (blue diamond). Statistical analysis comparing mutant LDT values to the wild type was performed on the slope of the lesion size curve during the spreading phase before LDT values were derived. Asterisks indicate significance levels: $p < 0.05$ (*), $p < 0.01$ (**), $p < 0.001$ (***), ns = no significant difference with the wild-type. **B**. The lesion doubling time (LDT) was measured and analyzed under the same conditions as in A for Columbia-3 (Col-3), the wild-type genotype, and a mutant corresponding to one gene. Measurements were obtained from n = 20 to 27 leaves across two to three experiments (see Table in C). **C**. The table shows mutant names, the corresponding Nottingham Arabidopsis Stock Centre (NASC) number, the gene with its associated T-DNA insertion, the genetic background, the T-DNA insertion location as provided by TAIR, the mean LDT, the number of experiments and leaves per mutant, and the p-value obtained using a linear mixed-effects model to analyze the phenotypic response to *S. sclerotiorum*. The model included 'Line' as a fixed effect and 'Experiment' as a random effect: slope ~ genotype + (1 | experiment) (lmer function, lme4 package in R). Homozygous status for T-DNA insertion was genotyped by PCR using forward and reverse primers, along with the corresponding primers LB1_SAIL or LBb1.3_SALK for SALK or SAIL mutants.
